# Risk of adverse outcomes during gabapentinoid therapy and factors associated with increased risk in UK primary care using the clinical practice research datalink: a cohort study

**DOI:** 10.1097/j.pain.0000000000003239

**Published:** 2024-04-11

**Authors:** Sara Muller, James Bailey, Ram Bajpai, Toby Helliwell, Sarah A. Harrisson, Rebecca Whittle, Christian D. Mallen, Julie Ashworth

**Affiliations:** a School of Medicine, Keele University, Keele, Staffordshire, United Kingdom; b Midlands Partnership University Foundation Trust, Stafford, Staffordshire, United Kingdom; c Institute of Applied Health Research, College of Medical and Dental Sciences, University of Birmingham, Birmingham, United Kingdom

**Keywords:** Gabapentinoids, Primary care, Adverse events

## Abstract

Growing evidence from pharmacovigilance data and postmortem toxicology reports highlights the misuse potential of gabapentinoids. This study aimed to investigate the risk of serious adverse outcomes (drug misuse, overdose, major trauma), and their risk factors, in primary care patients who are prescribed gabapentinoids. Using the UK Clinical Practice Research Datalink, a matched cohort study calculated adverse event rates separately for gabapentinoid-exposed and unexposed cohorts. In the exposed cohort, event rates for exposure to a range of potential risk factors were calculated. Event rates were compared using Cox proportional hazards models, adjusted for age, sex, deprivation, previous mental health diagnosis, and coprescribing with potentially interacting medicines. Substance misuse (gabapentin adjusted hazard ratio [95% CI]: 2.40 [2.25-2.55]), overdose (2.99 [2.56-3.49]), and major trauma (0-2.5 years: 1.35 [1.28-1.42]; 2.5 to 10 years: 1.73 [1.56-1.95]) were more common among patients prescribed gabapentinoids than matched individuals who were not. The association with overdose was stronger for pregabalin than gabapentin. All adverse outcomes were significantly associated with smoking, history of substance misuse, overdose, or a mental health condition and prescription of opioids, benzodiazepines, antidepressants, and Z-drug hypnotics (eg, gabapentin hazard ratios for association of concurrent opioid use: misuse 1.49 [1.47-1.51]; overdose 1.87 [1.78-1.96]; major trauma 1.28 [1.26-1.30]). Our findings highlight the importance of careful patient selection when prescribing gabapentinoids and the need to educate prescribers about the risks of these drugs, particularly in combination with other central nervous system depressants.

## Abstract 2

Gabapentinoids are commonly prescribed medicines. We show they are associated serious adverse events, highlighting the importance of careful patient selection when considering gabapentinoid therapy.

## 1. Introduction

Gabapentin and pregabalin (gabapentinoids) are anticonvulsants drugs that are approved in the United Kingdom and European Union to treat epilepsy, neuropathic pain, and, in the case of pregabalin, generalised anxiety disorder. During the last 2 decades, gabapentinoid prescribing has risen markedly in the United Kingdom and globally.^2,4^ Pain is the commonest reason for prescribing, and there is widespread off-label prescribing for non-neuropathic pain, despite a lack of evidence to support this.^2,17^ In the United Kingdom, it has been estimated that over 50% of gabapentinoid prescriptions were for unlicensed indications.^2^ Gabapentinoids were initially considered as safe medicines and nonaddictive, but a growing body of evidence has highlighted their potential to cause harm.^10,12^

Around 60% of patients experience central nervous system (CNS) depressant side effects with gabapentinoids, including drowsiness, dizziness, and ataxia,^8,28^ which may increase the risk of falls leading to injury and fractures.^13^ In addition, gabapentinoids are frequently coprescribed with other CNS depressants including opioids and benzodiazepines,^2^ which may potentiate their respiratory depressant effect and further increase the risk of falls, especially in older people.^5^ Growing evidence has highlighted the misuse potential of gabapentinoids.^10,12^ An international adverse event database identified almost 12,000 reports of gabapentinoid abuse from 2004 to 2015, with >75% reported since 2012.^6^ There has also been a rise in gabapentinoid-related deaths, with UK deaths involving gabapentinoids increasing from 12 in 2012 to 170 in 2016.^6,19,25^ Most gabapentinoid-related deaths involve other psychoactive and sedative drugs, with up to 90% also involving opioids.^3,6,10,19,22^ Pregabalin has been reported to have a higher misuse potential than gabapentin, which is thought to be due to its more rapid absorption and faster onset of action.^3,19^

Concerns about rising use and misuse led to gabapentin and pregabalin being reclassified in the United Kingdom as scheduled 3 (class C) controlled drugs from April 2019,^24^ and thereafter, gabapentinoid prescribing has been subject to stricter controls. However, reports of gabapentinoid misuse and addiction typically involve people with a history of substance abuse, most commonly opioids such as heroin or methadone,^10^ and therefore, the generalisability of the published evidence regarding the risks of gabapentinoids to the general patient population is currently limited.^3^ There is an argument that people without a substance abuse history are at low risk of gabapentinoid misuse, and there are few reported cases in this population.^10,12^ However, no epidemiological studies have investigated the prevalence of gabapentinoid misuse in people without a substance misuse history.^3^ To address these evidence gaps, this study investigates the risks of gabapentinoids, and explores factors associated with increased risk, in a large sample of primary care patients who are prescribed gabapentinoids. Specifically, this study aims to describe (1) the risk of serious adverse outcomes (overdose, drug misuse, and major trauma) in primary care patients who are prescribed a gabapentinoid compared with those who are not and (2) the potential risk factors associated with gabapentinoid users experiencing an adverse outcome.

## 2. Methods

This was an observational database study performed in the Clinical Practice Research Datalink (CPRD) Aurum (December 2019), linked to Hospital Episode Statistics (HES) Admitted Patient Care and Outpatient datasets, and individual-level Indices of Multiple Deprivation (IMD). CPRD Aurum is high-quality database of deidentified coded primary care records that captures diagnoses, symptoms, prescriptions, referrals, and tests from 1082 general practices across England. It is considered to be broadly representative of the English population in terms of age, sex, and ethnicity.^30^ The Independent Scientific Advisory Committee (ISAC) provided permission for data access (ISAC protocol 19_214A). Details of all definitions are explained in the Supplementary Materials (http://links.lww.com/PAIN/C36). All clinical and drug code lists were established by a clinical academic pain specialist and an academic general practitioner and are freely available from the Keele University repository (http://doi.org/10.21252/k6bc-ys67).

### 2.1. Matched cohort study

#### 2.1.1. Exposed cohort—users of gabapentinoids

We defined an individual as a user of gabapentinoids if they had a record of a gabapentin or pregabalin prescription in CPRD Aurum between April 1997 (gabapentin)/April 2004 (pregabalin) and October 2018, and this was on or after their 18th birthday. These dates were chosen to reflect the licensing of the drugs for neuropathic pain in the United Kingdom and the availability of linked datasets at the time of the CPRD Aurum data download. All individuals in the exposed cohort were assigned an index date—the date of their first gabapentinoid prescription—and were followed up until the end of their first gabapentinoid treatment period or the date the patient no longer contributed data (due to leaving the practice, the practice leaving CPRD, or patient death), whichever occurred earlier.

Where an individual had multiple treatment periods with the same drug, only the first period was included in analyses. Should an individual have received both gabapentin and pregabalin, they were included in both analyses. As all analyses of gabapentin and pregabalin were conducted separately, this does not result in “double counting” of any individual and all observations remain independent within each analysis.

#### 2.1.2. Unexposed cohort

Individuals in the exposed cohort were matched based on year of birth, sex, general practice, and follow-up time (within 365 days) to another individual who had no record of a gabapentinoid prescription. Individuals in the unexposed cohort were assigned the same index date as their matched exposed individual and allocated an end of treatment date at random based on the distribution of follow-up times in the exposed cohort.

In both the exposed and unexposed cohorts, individuals were required to have been contributing data for at least 1 year before their index date and were followed up until the earliest of the end of the first treatment period (proxy date for unexposed), the first outcome of interest, the end of the patient's record, or 10 years after the index date (where the treatment period exceeded 10 years).

#### 2.1.3. Outcomes of interest

Three potential adverse outcomes were considered: major trauma, drug misuse, and overdose. Major trauma, defined as bone fracture, joint dislocation, ligament or tendon rupture, or head trauma (including subdural haemorrhage), is a plausible adverse event given the side-effect profile of gabapentinoids,^13^ and there is previous evidence of gabapentinoid-related overdose and misuse in selected high-risk populations. As most reported gabapentinoid-related overdoses also include other drugs, overdose (deliberate or accidental) of any substance, not specifically a gabapentinoid, was included. All outcomes were defined in CPRD Aurum, HES Admitted Patient Care, and HES Outpatients datasets. The first adverse event for each individual, across the 3 datasets, was considered an outcome event.

#### 2.1.4. Statistical analyses

The absolute rate of each adverse event per 10,000 person-years was calculated separately in the exposed and unexposed cohorts. Rates of events were compared between the cohorts using Cox proportional hazards models, unadjusted and adjusting for age, sex, IMD quintile, previous mental health diagnoses (anxiety, depression, bipolar disorder, schizophrenia, and psychosis), and concurrent prescription of antidepressants, benzodiazepines, Z-drug hypnotics (zopiclone or zolpidem), and opioids at the index date. In models with drug misuse as the outcome, individuals with a recorded history of drug misuse were excluded, as this can be considered a chronic relapsing condition.^1^ In models with major trauma and overdose as the outcomes, adjustment was made for history of major trauma and overdose, respectively, as these were considered acute events. The appropriateness of the proportional hazards assumption was assessed graphically and, where necessary, time-varying covariates included in the model. Where the assumption of proportional hazards was violated for the exposure of gabapentinoid use, an interaction with time was fitted directly and a hazard ratio was presented for each time period. Robust standard errors were used due to the matched nature of the cohort. The results were presented as hazard ratios (HR) with 95% confidence intervals (95% CI). Follow-up time was censored at the earliest of the first treatment period, the first outcome of interest, the end of the patient's record, or 10 years after the index date.

### 2.2. Sample size considerations

Assuming the annual incidence of trauma of around 0.02%,^18^ 1:1 matching with our sample size will give us more than 82% power to detect a hazard ratio of 2.2 at 5% level of significance. For our drug misuse outcome, assuming the annual event rate of 0.15%,^14^ we will have more than 91% power to detect a hazard ratio of 1.4 at 5% significance level. Finally, assuming an annual incidence of overdose of 0.2% (based on a feasibility study), we will have more than 85% power to detect a HR of 1.3.

### 2.3. Risk factor study

#### 2.3.1. Exposures

Using only the exposed group from the matched-cohort study, exposures were defined as age group at index date (18-24, 25-34, 35-44, 45-54, 55-64, 65-74, ≥75 years), sex, and IMD quintile (quintile 1 is least deprived), smoking status (current, ex, never smoker), body mass index (BMI) before index date (underweight <18.5 kg/m^2^; 18.5 kg/m^2^ ≤ normal <25 mg/m^2^; 25 kgm^2^ ≤ overweight < 30 kg/m^2^; 30 kg/m^2^ ≤ obese), gabapentinoid dose in mg (starting, average, and maximum during the first treatment period, modelled in quintiles of the sample distribution due to skewness), history of substance misuse, mental health disorders, previous overdose, and coprescription of an antidepressant, benzodiazepine, Z-drug hypnotic, or opioid. All treatment periods were assumed to have started when the prescription was issued. The duration of a prescription was calculated based on information provided in the prescription, giving each an end date. A coprescription with a gabapentinoid was defined when there was an overlap of the prescription periods of the gabapentinoid and the other drug, based on the start and end dates. We have previously described this process in detail.^2^

Where a prescription or diagnosis was recorded, it was assumed to have occurred and if not recorded was assumed not to have occurred. BMI and smoking status were missing in <7% and <1.5% of individuals, respectively, and are unlikely to be missing at random. Individuals with missing values were therefore excluded from analysis of these variables.

#### 2.3.2. Outcomes

Outcomes were major trauma, drug misuse, and overdose, as defined above.

#### 2.3.3. Statistical analyses

The absolute rate of each adverse event per 10,000 person-years was calculated separately in the different exposure groups. Rates of events were compared between exposure groups using Cox proportional hazards models, adjusted for age, sex, and IMD quintile. In models with drug misuse as the outcome, individuals with a recorded history of drug misuse were excluded.^1^ In models with overdose as the outcome, adjustment was made for a history of overdose. The appropriateness of the proportional hazards assumption was assessed graphically and, where necessary, time-varying covariates included in the model. The results were presented as hazard ratios (HR) with 95% confidence intervals (95% CI).

All analyses were conducted in Stata 14.0 or above,^29^ and gabapentin and pregabalin were considered separately.

## 3. Results

During the study period, 481,046 people with an incident gabapentin prescription and 256,860 with an incident pregabalin prescription contributed data to CPRD Aurum, and their records could be linked to HES data. Those with incident gabapentin prescriptions contributed a total of 2,285,102 person-years of follow-up (median 3.8 years per person). Mean age was 58.5 years (standard deviation 16.5), and 61.5% were female patients. Those with incident pregabalin prescriptions had a total of 1,105,475 person-years of follow-up (median 3.4 years per person). Mean age was 57.7 (16.9) years, and 62.6% were female females.

### 3.1. Matched cohort

Of the individuals with gabapentin and pregabalin prescriptions, 391,655 and 226,685, respectively, were successfully matched to an individual without a gabapentinoid prescription. Substance misuse and overdose were significantly more common during a first gabapentinoid treatment period than in matched unexposed individuals who were never prescribed a gabapentinoid (Table 1). For substance misuse, the association was similar for gabapentin and pregabalin (unadjusted hazard ratio [95% CI] gabapentin 3.13 [2.96-3.30], pregabalin 3.32 [3.12-3.52]), but the association with overdose was stronger for pregabalin (8.71 [7.51-10.11] vs 4.96 [4.33-5.67] for gabapentin). Associations were attenuated by adjustment for demographic factors, previous mental health conditions, and concurrent use of potentially interacting medications but remained significant (adjusted hazard ratio [95% CI] for substance misuse; gabapentin 2.40 [2.25-2.55], pregabalin 2.41 [2.25-2.58], and for overdose; pregabalin 4.09 [3.47-4.82] vs 2.99 [2.56-3.49] for gabapentin).

**Table 1:** Matched cohort study: association between gabapentinoid use and adverse outcomes.

Gabapentin	Rate per 10,000 person-years		Hazard ratio (95% CI)	
	Exposed (n = 391,655)	Unexposed (n = 391,655)	Unadjusted	Adjusted*
Misuse	233.0 (226.6-239.5)	74.3 (70.8-77.9)	3.13 (2.96-3.30)	2.40 (2.25-2.55)
Overdose	51.6 (48.8-54.5)	10.4 (9.2-11.8)	4.96 (4.33-5.67)	2.99 (2.56-3.49)
Major trauma				
0-2.5 y	222.0 (215.1-229.0)	219.1 (212.4-226.2)	1.01 (0.97-1.06)	1.35 (1.28-1.42)
2.5-10 y	195.9 (184.6-208.0)	107.6 (99.2-116.7)	1.82 (1.65-2.01)	1.73 (1.56-1.92)

Pregabalin	Rate per 10,000 person-years		Hazard ratio (95% CI)	
	Exposed (n = 226,685)	Unexposed (n = 226,685)	Unadjusted	Adjusted*
Misuse	260.2 (252.7-267.9)	78.2 (73.8-81.9)	3.32 (3.12-3.52)	2.41 (2.25-2.58)
Overdose	85.8 (81.7-90.0)	9.8 (8.5-11.3)	8.71 (7.51-10.11)	4.09 (3.47-4.82)
Major trauma				
0-2.5 y	243.9 (235.3-252.8)	221.8 (213.6-230.3)	1.10 (1.04-1.16)	1.36 (1.28-1.44)
2.5-10 y	195.2 (185.0-206.0)	113.4 (105.7-121.8)	1.72 (1.57-1.88)	1.63 (1.48-1.79)

Previous trauma and overdose when these were the outcomes of interest.

Misuse analysis in those with no history of misuse only.

* Adjusted for age, sex, index of multiple deprivation rank quintiles, previous mental health diagnosis, concurrent use of antidepressants, benzodiazepines, nonbenzodiazepine hypnotics (Z-drugs), opioids.

Less strong associations were seen between gabapentinoid treatment and major trauma (Table 1). It was necessary to fit an interaction with time (ie, the association between gabapentinoid treatment and trauma was not constant over time), with a stronger association later in the treatment period (after 2.5 years). Adjustment for mental health conditions and concurrent, potentially interacting prescriptions strengthened the association between gabapentinoid prescription and trauma in the first 2 and a half years of the treatment period.

### 3.2. Risk factor study

Rates of misuse and overdose were higher at younger ages and in those in the most deprived areas for gabapentin (Table 2) and pregabalin (Table 3) (unadjusted estimates are shown in Supplementary Table 1, http://links.lww.com/PAIN/C36). Rates of trauma were higher at older ages, in male patients, and in those living in less deprived neighbourhoods for both drugs. After adjusting for age, sex, IMD, history of mental health conditions, and concurrent potentially interacting prescriptions, all adverse outcomes during pregabalin and gabapentin use were significantly associated with BMI, with lower rates of events in those who were overweight and obese compared with those who were normal/underweight. Current smoking conferred a significantly higher risk of all adverse events compared with never or previously smoking. History of substance misuse, overdose, or a mental health condition and prescription of potentially interacting medications were all associated with an increased risk of all adverse outcomes. There was little pattern of association between dose of gabapentin and any of the outcomes, other than an increased risk of all outcomes in the highest quintile of starting, average, and maximum doses. For pregabalin, associations were similar, but there was a stronger indication of a potential dose-dependent association with misuse and overdose.

**Table 2:** Risk factor study—gabapentin: association between potential risk factors and adverse outcomes.

Characteristics (n)	Misuse		Overdose		Trauma	
	Rate per 10,000 person-years (95% CI)	Hazard ratio* (95% CI)	Rate per 10,000 person-years (95% CI)	Hazard ratio* (95% CI)	Rate per 10,000 person-years (95% CI)	Hazard ratio* (95% CI)
Age group (y)						
18-25	632.5 (604.4-661.9)		105.9 (95.4-117.7)		272.8 (255.0-297.9)	
25-34	610.3 (596.4-624.6)		85.8 (80.9-90.9)		221.1 (213.1-229.5)	
35-44	473.4 (465.5-481.5)		61.9 (59.3-64.7)		205.1 (200.1-210.3)	
45-54	443.5 (437.1-450.0)		42.8 (41.0-44.8)		226.8 (222.4-231.4)	
55-64	363.6 (357.8-369.4)		26.2 (24.8-27.8)		277.6 (272.6-282.7)	
65-74	266.5 (261.3-271.7)		23.5 (22.0-25.1)		391.6 (385.2-398.0)	
≥75	164.9 (160.5-169.5)		29.5 (27.7-31.4)		812.8 (802.2-823.5)	
Sex						
Female	429.0 (424.3-433.8)		40.0 (38.6-41.4)		310.2 (306.2-314.3)	
Male	327.1 (323.9-330.3)		40.3 (39.2-41.4)		372.5 (369.1-376.0)	
IMD fifth						
1 (least deprived)	203.4 (198.9-208.0)		26.9 (25.3-28.5)		366.3 (360.1-372.6)	
2	263.7 (258.6-268.9)		31.5 (29.8-33.3)		359.7 (353.6-365.8)	
3	329.5 (323.7-335.4)		37.3 (35.4-39.2)		352.6 (346.6-358.7)	
4	421.8 (415.4-428.4)		43.1 (41.2-45.1)		335.6 (330.0-341.4)	
5 (most deprived)	580.3 (573.0-587.6)		58.4 (56.3-60.6)		334.5 (329.2-340.0)	
Body mass index†						
Normal/underweight	443.6 (438.0-449.3)	1	45.8 (44.1-47.5)	1	396.8 (391.5-402.1)	1
Overweight	338.9 (334.4-343.5)	0.72 (0.71-0.73)	39.7 (38.2-41.2)	0.91 (0.87-0.96)	317.8 (313.4-322.2)	0.82 (0.80-0.83)
Obese	334.4 (329.9-339.0)	0.77 (0.76-0.79)	34.7 (33.4-36.2)	0.85 (0.81-0.90)	348.9 (344.3-353.6)	0.84 (0.82-0.86)
Smoking status‡						
Current	1076.2 (1067.0-1085.5)	1	64.2 (62.2-66.2)	1	314.4 (309.8-319.1)	1
Never	61.0 (59.4-62.6)	0.07 (0.07-0.07)	28.4 (27.3-29.6)	0.54 (0.51-0.57)	347.1 (343.1-351.2)	0.88 (0.86-0.89)
Previous	242.0 (237.8-246.2)	0.26 (0.26-0.27)	32.7 (31.2-34.2)	0.66 (0.62-0.70)	392.6 (387.2-398.1)	0.96 (0.94-0.98)
History of substance misuse	NA					
No			40.0 (39.2-40.9)	1	648.8 (346.2-351.5)	1
Yes			138.5 (105.0-182.7)	1.99 (1.51-2.63)	368.6 (309.3-439.2)	1.55 (1.30-1.84)
History of overdose§						
No	358.4 (335.8-361.1)	1	38.1 (37.3-39.0)	1	348.0 (345.4-350.7)	1
Yes	1214.1 (1158.6-1272.3)	2.54 (2.42-2.66)	281.7 (258.0-307.6)	5.58 (5.09-6.12)	443.9 (413.3-476.8)	1.63 (1.52-1.75)
History of mental health condition						
No	297.2 (294.4-300.1)	1	27.9 (27.1-28.8)	1	340.3 (337.2-343.4)	1
Yes	536.3 (530.2-542.5)	1.68 (1.66-1.71)	68.8 (67.8-72.0)	2.31 (2.21-2.42)	369.6 (364.6-374.6)	1.22 (1.20-1.25)
Benzodiazepines‖						
No	342.2 (339.4-345.0)	1	32.9 (32.1-33.8)	1	340.0 (337.3-342.8)	1
Yes	528.5 (519.3-537.8)	1.55 (1.52-1.58)	90.6 (87.0-94.3)	2.64 (2.51-2.77)	410.4 (402.4-418.5)	1.26 (1.23-1.28)
Nonbenzodiazepine hypnotics‖						
No	348.7 (346.0-351.4)	1	35.3 (34.5-36.1)	1	342.5 (339.8-345.1)	1
Yes	341.3 (626.3-656.7)	1.81 (1.76-1.85)	119.7 (113.7-126.0)	3.21 (3.03-3.40)	453.3 (441.0-466.0)	1.34 (1.30-1.38)
Antidepressants‖						
No	295.5 (292.3-298.7)	1	61.9 (60.3-63.6)	1	332.8 (329.4-336.2)	1
Yes	460.6 (456.0-465.3)	1.49 (1.47-1.51)	23.9 (23.0-24.8)	2.46 (2.35-2.57)	370.4 (366.3-374.5)	1.22 (1.20-1.24)
Opioids‖						
No	288.7 (285.2-292.2)	1	50.8 (49.5-52.1)	1	297.0 (293.5-30.6)	1
Yes	431.0 (427.0-435.0)	1.49 (1.47-1.51)	27.5 (26.5-28.6)	1.87 (1.78-1.96)	393.2 (389.5-397.1)	1.28 (1.26-1.30)
Gabapentin starting dose (mg, fifths)						
1 (100-300)	343.7 (338.4-349.1)	1	38.0 (36.3-39.6)	1	377.5 (371.9-383.1)	1
2 (300.01-450)	350.2 (343.3-357.3)	1.02 (1.00-1.05)	33.7 (31.6-35.8)	0.89 (0.83-0.97)	364.8 (357.7-372.0)	0.98 (0.96-1.01)
3 (450.01-900)	375.9 (370.9-381.0)	1.07 (1.05-1.10)	42.5 (40.9-44.2)	1.11 (1.05-1.18)	343.6 (338.8-348.5)	1.02 (1.00-1.04)
4 (900.01-1071.4)	423.0 (414.2-341.9)	1.13 (1.10-1.16)	35.3 (32.9-37.8)	0.88 (0.81-0.96)	320.3 (312.7-328.0)	0.98 (0.96-1.01)
5 (1071.41-4800)	354.5 (348.9-360.1)	1.05 (1.03-1.07)	45.8 (43.9-47.8)	1.23 (1.16-1.31)	329.5 (324.2-335.0)	1.03 (1.01-1.05)
Gabapentin average dose (mg, fifths)						
1 (100-300)	332.8 (326.9-338.7)	1	36.0 (34.2-37.9)	1	376.7 (370.4-383.0)	1
2 (300.01-600)	343.4 (337.5-349.4)	1.05 (1.02-1.07)	36.9 (35.1-38.9)	1.05 (0.97-1.13)	375.9 (369.7-382.2)	1.1 (0.99-1.03)
3 (600.01-900)	367.3 (361.5-373.2)	1.09 (1.06-1.11)	40.5 (38.7-42.5)	1.12 (1.05-1.20)	337.9 (332.3-343.6)	1.1 (0.98-1.03)
4 (900.01-1261.4)	370.1 (364.4-376.0)	1.09 (1.07-1.12)	36.9 (35.1-38.7)	1.02 (0.95-1.10)	328.1 (322.7-333.6)	1.00 (0.98-1.03)
5 (1261.41-4800)	409.9 (406.4-416.5)	1.19 (1.16-1.21)	50.8 (48.6-53.0)	1.37 (1.28-1.46)	330.5 (324.7-336.4)	1.08 (1.05-1.11)
Gabapentin maximum dose (mg, fifths)						
1 (100-303.1)	334.7 (328.8-340.7)	1	36.0 (34.2-38.0)	1	370.7 (364.4-377.0)	1
2 (300.11-706.4)	337.1 (331.2-343.1)	1.03 (1.01-1.06)	36.4 (34.6-38.4)	1.04 (0.96-1.12)	371.1 (364.8-377.4)	1.01 (0.99-1.04)
3 (706.5-1047.6)	371.3 (365.3-377.4)	1.09 (1.06-1.12)	40.4 (38.6-42.4)	1.12 (1.04-1.20)	335.0 (329.3-340.8)	1.01 (0.99-1.04)
4 (1047.7-1542.9)	360.8 (355.0-366.8)	1.07 (1.04-1.10)	37.1 (35.3-39.0)	1.03 (0.96-1.11)	329.9 (324.3-335.6)	1.01 (0.99-1.04)
5 (1542.91-4800)	412.4 (406.3-418.6)	1.20 (1.17-1.23)	49.5 (47.5-51.5)	1.35 (1.26-1.44)	342.1 (336.6-347.7)	1.10 (1.07-1.12)

* Adjusted for age, sex, IMD, history of overdose.

§ Not adjusted for history of overdose.

† Missing for n = 33,480, 6.96%.

‡ Missing for n = 7,101, 1.48%.

‖ Drug use is concurrent with gabapentin.

IMD, Indices of Multiple Deprivation.

**Table 3:** Risk factor study—pregabalin: association between potential risk factors and adverse outcomes.

Characteristics (n)	Misuse		Overdose		Trauma	
	Rate per 10,000 person-years (95% CI)	Hazard ratio* (95% CI)	Rate per 10,000 person-years (95% CI)	Hazard ratio* (95% CI)	Rate per 10,000 person-years (95% CI)	Hazard ratio* (95% CI)
Age group (y)						
18-25	681.1 (643.0-721.5)		205.4 (185.8-227.0)		258.0 (235.7-282.4)	
25-34	773.9 (752.4-796.0)		145.2 (136.6-154.2)		234.6 (223.4-246.2)	
35-44	593.4 (581.0-606.2)		89.1 (84.6-93.8)		218.0 (210.8-225.5)	
45-54	524.9 (515.2-534.9)		61.8 (58.7-65.1)		242.7 (236.3-249.3)	
55-64	429.6 (720.5-438.7)		40.7 (38.1-43.5)		298.7 (291.3-306.3)	
65-74	307.7 (299.6-316.0)		33.2 (30.7-35.9)		429.6 (419.8-439.5)	
≥75	189.0 (182.1-196.1)		40.0 (36.9-43.3)		885.1 (869.2-901.3)	
Sex						
Female	515.6 (508.1-523.3)		62.7 (60.3-65.3)		331.7 (325.8-337.8)	
Male	400.6 (395.6-405.7		61.1 (59.2-63.0)		392.7 (387.8-397.7)	
IMD fifth*						
1 (least deprived)	548.9 (242.0-256.1)		43.6 (40.8-46.6)		385.6 (376.8-394.7)	
2	320.6 (312.6-328.9)		49.6 (46.6-52.8)		382.4 (373.5-391.5)	
3	394.5 (385.5-403.7)		57.2 (54.0-60.7)		371.8 (363.1-380.7)	
4	514.2 (504.1-524.4)		68.7 (65.3-72.3)		358.8 (350.6-367.3)	
5 (most deprived)	709.3 (697.8-720.9)		84.7 (81.1-88.5)		355.7 (347.9-363.6)	
Body mass index†						
Normal/underweight	536.7 (528.1-545.4)	1	73.3 (70.3-76.4)	1	423.6 (416.1-431.3)	1
Overweight	396.0 (389.1-403.1)	0.70 (0.69-0.72)	58.4 (55.9-61.0)	0.87 (0.81-0.92)	335.5 (329.2-342.0)	0.80 (0.78-0.83)
Obese	399.6 (392.5-406.8)	0.77 (0.75-0.79)	52.3 (49.9-54.8)	0.83 (0.78-0.88)	366.2 (359.4-373.0)	0.82 (0.80-0.84)
Smoking status‡						
Current	1330.6 (1315.9-1345.5)	1	102.9 (99.3-106.5)	1	333.1 (326.5-339.8)	1
Never	77.4 (74.9-80.0)	0.07 (0.07-0.07)	42.5 (40.7-44.5)	0.52 (0.49-0.55)	368.5 (362.8-374.4)	0.87 (0.84-0.89)
Previous	279.1 (279.6-292.6)	0.26 (0.25-0.26)	47.5 (45.0-50.1)	0.61 (0.57-0.65)	416.8 (409.0-424.8)	0.94 (0.81-0.97)
History of substance misuse	NA					
No			61.2 (59.7-62.7)	1	370.2 (366.4-374.1)	1
Yes			265.8 (208.8-338.4)	1.95 (1.53-2.50)	362.0 (293.4-446.7)	1.44 (1.16-1.77)
History of overdose§						
No	431.5 (427.4-435.7)	1	57.5 (56.1-59.0)	1	369.0 (365.2-372.9)	1
Yes	1548.1 (1468.2-1632.3)	2.60 (2.46-2.74)	452.7 (414.5-494.3)	5.58 (5.09-6.13)	473.3 (434.2-515.9)	1.64 (1.50-1.79)
History of mental health condition						
No	348.9 (344.3-353.5)	1	39.0 (37.6-40.5)	1	361.1 (356.5-365.8)	1
Yes	628.4 (619.7-637.2)	1.62 (1.59-1.65)	104.9 (101.6-108.3)	2.40 (2.28-2.52)	387.4 (380.8-394.2)	1.22 (1.19-1.25)
Benzodiazepines‖						
No	397.4 (393.0-401.8)	1	45.7 (44.3-47.2)	1	356.3 (352.0-360.3)	1
Yes	675.3 (662.4-688.5)	1.65 (1.61-1.69)	141.1 (135.6-146.7)	2.84 (2.70-2.99)	440.3 (430.2-450.6)	1.31 (1.28-1.35)
Nonbenzodiazepine hypnotics‖						
No	410.2 (406.0-414.5)	1	50.0 (48.6-51.4)	1	360.3 (356.4-364.3)	1
Yes	793.4 (773.96-813.2)	1.82 (1.77-1.87)	183.1 (174.5-192.0)	3.27 (3.09-3.46)	472.0 (457.7-486.8)	1.37 (1.32-1.41)
Antidepressants‖						
No	337.7 (332.7-342.9)	1	32.5 (31.0-34.0)	1	351.6 (346.4-356.8)	1
Yes	556.8 (549.9-563.7)	1.50 (1.47-1.53)	92.6 (90.0-95.3)	2.54 (2.40-2.68)	389.9 (384.3-395.6)	1.26 (1.23-1.29)
Opioids‖						
No	345.5 (340.0-351.0)	1	52.8 (50.7-54.9)	1	312.1 (307.0-317.4)	1
Yes	523.3 (517.1-529.6)	1.53 (1.50-1.56)	68.9 (66.8-71.0)	1.38 (1.31-1.45)	418.2 (412.7-423.8)	1.29 (1.27-1.32)
Pregabalin starting dose (mg, fifths)						
1 (50-75)	378.7 (370.2-387.4)	1	51.7 (48.7-54.8)	1	434.1 (425.0-443.5)	1
2 (75-147)	436.9 (426.5-447.5)	1.10 (1.07-1.14)	57.4 (53.9-61.2)	1.08 (0.99-1.17)	396.0 (386.1-406.1)	0.99 (0.95-1.02)
3 (147-150)	432.2 (424.6-440.0)	1.06 (1.03-1.09)	53.0 (50.5-55.7)	1.00 (0.92-1.08)	335.6 (329.0-342.4)	0.93 (0.91-0.96)
4 (150-225)	427.6 (417.0-438.4)	1.09 (1.05-1.12)	61.0 (57.2-65.0)	1.17 (1.07-1.28)	346.2 (336.8-355.9)	0.95 (0.92-0.99)
5 (225-600)	541.5 (530.9-552.4)	1.28 (1.24-1.32)	89.5 (85.5-93.7)	1.59 (1.48-1.72)	354.4 (346.0,363.0)	1.06 (1.02-1.09)
Pregabalin average dose (mg, fifths)						
1 (50-88)	363.8 (354.8-343.0)	1	52.0 (48.7-55.4)	1	450.1 (440.0-460.5)	1
2 (88-150)	405.7 (398.9-412.5)	1.04 (1.01-1.07)	49.4 (47.2-51.8)	0.92 (0.85-1.00)	353.7 (347.4-360.0)	0.92 (0.89-0.94)
3 (150-170)	447.2 (427.4-467.9)	1.14 (1.09-1.20)	63.1 (56.3-70.9)	1.17 (1.03-1.34)	384.6 (366.3-403.7)	0.97 (0.92-1.02)
4 (170-300)	437.4 (429.0-446.0)	1.12 (1.08-1.16)	64.6 (61.6-67.8)	1.20 (1.10-1.30)	350.0 (342.4-357.6)	0.96 (0.93-1.00)
5 (300-600)	587.5 (576.4-598.9)	1.37 (1.33-1.42)	88.1 (84.1-92.2)	1.49 (1.38-1.62)	351.9 (343.5-360.5)	1.10 (1.07-1.14)
Pregabalin maximum dose (mg, fifths)						
1 (50-100)	374.1 (366.0-382.5)	1	50.7 (47.8-53.7)	1		1
2 (100-150)	402.6 (395.0-410.5)	1.2 (1.00-1.06)	49.7 (47.2-52.4)	0.97 (0.90-1.05)	427.2 (418.4-436.2)	0.92 (0.89-0.94)
3 (150-200)	388.8 (376.3-401.6)	1.2 (0.98-1.07)	52.0 (47.7-56.6)	1.03 (0.93-1.14)	341.8 (334.8-349.0)	0.95 (0.91-0.98)
4 (200-400)	458.1 (448.8-467.7)	1.16 (1.13-1.20)	70.3 (66.9-74.0)	1.35 (1.25-1.46)	357.7 (345.8-370.0)	0.99 (0.96-1.02)
5 (400-600)	574.5 (564.1-585.2)	1.36-(1.32-1.40)	83.7 (80.0-87.6)	1.49 (1.38-1.60)	360.2 9352.0-368.7)	1.12 (1.09-1.16)

* Adjusted for age, sex, and IMD.

† Missing for n = 15,444, 6.01%.

‡ Missing for n = 1,440, 0.56%.

§ Not adjusted for history of overdose.

‖ Drug use is concurrent with gabapentin.

IMD, Indices of Multiple Deprivation.

## 4. Discussion

To our knowledge, this is the first study to examine the risk of serious adverse events, and the associated risk factors, in a primary care population prescribed gabapentinoids. We found higher rates of drug misuse, overdose, and major trauma in patients prescribed gabapentinoids compared with those who were not. There was no clear relationship between the dose of gabapentinoids and rates of adverse events, although slightly higher rates of adverse events were seen in the highest quintile of starting, average, and maximum dose. We identified several risk factors for these adverse events in patients prescribed gabapentinoids including current smoking, previous overdose, previous drug misuse, history of a mental health condition, and concurrent use of potentially interacting medication (opioids, benzodiazepines, Z-drug hypnotics, and antidepressants).

The published literature regarding the risks of gabapentinoids derives largely from pharmacovigilance data, postmortem toxicology databases, internet surveys, and case series, often in selected high-risk populations, limiting the scope for comparison with our primary care cohort. Our study found similar rates of recorded drug misuse among patients prescribed gabapentin and pregabalin, which was not expected. The pharmacokinetics of pregabalin, including faster onset, greater bioavailability, and higher potency compared with gabapentin, theoretically predispose to greater misuse potential, and in Europe, higher rates of misuse have previously been reported for pregabalin than gabapentin.^11^ However, in the United States, gabapentin misuse is more common, possibly because gabapentin prescribing is less heavily regulated in the United States.^8,28^ Previous studies found pregabalin was more likely to be implicated in postmortem toxicology reports than gabapentin,^3,10,12^ which is consistent with our finding that pregabalin prescription was more strongly associated with overdose. Our finding of higher rates of major trauma among patients prescribed gabapentinoids is consistent with the known side-effect profile of these drugs, including ataxia and dizziness,^8,28^ which may predispose to falls resulting in injury, and a published case report of falls associated with gabapentin therapy.^13^

The risk factors we identified for drug misuse and overdose are consistent with published observational studies using pharmacovigilance data, which found the highest rates of gabapentinoid misuse among people with a current or previous substance misuse disorder, particularly opioid misuse, and higher rates in those with psychiatric comorbidity.^10,12^ Coprescription of gabapentinoids with opioids, benzodiazepines, antidepressants, or Z-drug hypnotics also increased the risk of serious adverse events, which was expected given their potentially synergistic CNS depressant effects.^6,19^ In particular, gabapentinoids can potentiate the respiratory depressant effects of opioids, which can increase the risk of opioid overdose.^15,16^ Published studies of postmortem toxicology reports indicate that the majority of overdoses involving gabapentinoids also involved opioids and, to a lesser extent, other CNS depressants.^15,16,26,31^ Previous observational studies reported an increased risk of fatal opioid overdose among opioid users coprescribed gabapentin or pregabalin,^26^ an increased risk of opioid and nonopioid overdose associated with coprescription of gabapentinoids and opioids,^5^ and an increased risk of overdose of any substance associated with coprescription of gabapentin with benzodiazepines.^26^ Lower rates of misuse and overdose were observed in those who were overweight and obese compared with those who had a normal or low BMI. There is no obvious pharmacokinetic explanation for this finding. Gabapentinoids are hydrophilic drugs, so excess adipose tissue is unlikely to substantially affect their distribution. The literature suggests a complex relationship between obesity and substance misuse, and while there may be some specific obese populations with higher rates of substance misuse, data generally indicate equal or even lower rates of substance misuse in the obese compared with the general population,^27^ which is consistent with our findings.

There is limited published evidence relating to the risk of traumatic injury associated with gabapentinoids. We found that history of mental health conditions, previous overdose, and substance misuse were all associated with increased risk of major trauma in patients prescribed gabapentinoids. This is consistent with published evidence indicating greater risk of falls and higher rates of injury among people with a range of mental health disorders.^23^ Coprescription of gabapentinoids with opioids, antidepressants, benzodiazepines, or Z-drug hypnotics increased the risk of major trauma, which is biologically plausible given their CNS depressant effects. A recent study reported a 69% higher rate of fall-related injury when gabapentinoids were added to existing opioid therapy, but not when a gabapentinoid and an opioid were started at the same time.^5^ The authors suggest that existing opioid use may be an effect modifier but acknowledge the possibility that residual confounding may explain the latter finding. Another study exploring opioid drug interactions associated with accidental trauma found a 40% higher rate of traumatic injury when pregabalin was coprescribed with the opioid oxycodone.^21^ Lower rates of major trauma were observed among patients prescribed gabapentinoids who were overweight or obese compared with those who had a normal or low BMI. Whilst this may seem surprising, it is consistent with published literature suggesting that, although obesity may be a risk factor for falls, low BMI (being underweight) is a risk factor for future fracture^7^ and for injurious falls in older people.^9^

A key strength of our study arises from the large size of the CPRD Aurum dataset, allowing analysis of a large cohort of primary care patients, representative of the broader population of England in terms of age, sex, and deprivation, with linked secondary care data. All prescribing is recorded electronically in UK primary care, so the likelihood of missing gabapentinoid prescribing data is low. Although some gabapentinoid prescribing could be initiated in hospital settings and first prescriptions not captured in this study, prescribing would be continued in primary care, so the likelihood of missing a whole treatment period is very low.

Our study has some limitations. Although we adjusted for age, sex, and deprivation score (and overdose/trauma when these were the outcomes); previous mental health conditions; and concurrent drug use and excluded patients with a history of drug misuse from models where drug misuse was the outcome, we still think there is likely to be residual confounding. Most gabapentinoid prescribing is for people with chronic pain,^2^ which may be a source of confounding by indication. However, chronic pain conditions are not reliably coded in a systematic way in UK primary health care records. Therefore, we did not limit inclusion in the study to those prescribed a gabapentinoid for pain. Depression and anxiety commonly co-occur with chronic pain.^20^ The relationship appears bidirectional, in that people with chronic pain are more likely to develop depression or anxiety and people with depression or anxiety are more likely to develop chronic pain.^20^ There is a similar bidirectional relationship between substance use disorders and chronic pain.^20^ Polypharmacy is also common in people with chronic pain, and gabapentinoids are frequently coprescribed with other CNS depressants including opioids, benzodiazepines, antidepressants, and hypnotics.^2^ Furthermore, pregabalin is licensed in the United Kingdom for the treatment of generalised anxiety disorder. Whilst we matched patients on key demographic variables and conditions that have been shown to be associated with increased risk in gabapentinoid use (mental health conditions and other CNS depressant drug use), these analyses are not intended to imply causality. We have previously shown that gabapentinoid prescribing is common in those with non-neuropathic pain conditions or where the indication for prescription is not clear. With this in mind, there will be residual confounding by indication in this heterogeneous patient population. Indeed, it may not be possible to delineate the reasons for gabapentinoid prescribing in any study design, as they are likely complex and multifactorial in many cases and subject to effect modification by a range of factors. In addition, we considered only the first of each outcome of interest during the first treatment period. It is likely that some people experienced multiple outcomes in this period, assuming that the outcome did not lead to the cessation of the drug. However, we do not think that inclusion of such multiple events would change the conclusions of this study.

In keeping with all database studies, our study is based on prescriptions issued and cannot account for over-the-counter medications or patients not taking their prescribed medication as directed, and the issue of a prescription does not necessarily mean the patient has taken the medication. In addition, some prescriptions lacked detail on dose prescribed or included improbable dosages, leading to assumptions.

Our findings of higher rates of drug misuse, overdose, and major trauma in a primary care population of patients prescribed gabapentinoids are concerning, given that these drugs are commonly prescribed in many countries worldwide.^4^ Much of this prescribing is “off label” and not supported by evidence of effectiveness.^2,17^ Furthermore, many people with chronic pain, for whom the majority of gabapentinoids are prescribed,^2^ may be at increased risk of gabapentinoid-related harm. Our findings highlight the importance of careful patient selection and the need to educate prescribers about the risks of combining gabapentinoids with other CNS depressant medication. Future research should focus on interventions and strategies to improve the appropriateness and safety of gabapentinoid prescribing.

## Conflict of interest statement

The authors have no conflicts of interest to declare.

## Supplemental video content

A video abstract associated with this article can be found on the PAIN Web site.

## Supplementary Material

SUPPLEMENTARY MATERIAL
